# Outcomes of Treating Symptomatic Intracranial Stenosis With Angioplasty or Stent Placement: A Retrospective Study in a Cohort of 37 Patients

**DOI:** 10.7759/cureus.91254

**Published:** 2025-08-29

**Authors:** Daniel Jaraki, Amber Dorn, Thomas Eckert, Roham Moftakhar

**Affiliations:** 1 Neurosurgery, University of South Carolina School of Medicine Columbia, Columbia, USA; 2 Neurosurgery, Prisma Health Midlands, Columbia, USA

**Keywords:** angioplasty, balloon angioplasty with stent, intracranial atherosclerotic disease, ischemic cerebrovascular disease, neurosurgery, retrospective research

## Abstract

Background

Intracranial atherosclerotic disease (ICAD) is a leading cause of ischemic stroke worldwide, with endovascular interventions such as angioplasty and stenting facing scrutiny due to variable safety outcomes in prior studies.

Objective

This study aimed to assess the safety of angioplasty with or without stenting for symptomatic ICAD and evaluate the predictors of procedural complications, including intervention timing, stenosis location, and clinical severity.

Method

In this retrospective analysis, 37 patients with symptomatic ICAD (70-99% stenosis) underwent angioplasty with stenting (n=32) or angioplasty alone (n=5). Patients were stratified into two groups: acute (≤7 days post-symptom onset) and semi-acute (>7 days post-symptom onset). Complications were assessed at 72 hours and 30 days.

Results

Technical revascularization succeeded in 36/37 cases (97.3%). Six patients (16.2%) experienced complications (all occurred in the acute group), including three deaths and three new neurological deficits within 72 hours. Angioplasty alone (all acute) had a 40% complication rate versus 19% with stenting (OR=0.35; p=0.29). Although numerically higher in acute versus subacute cases (28.6% vs. 0%), neither timing (p=0.16) nor posterior versus anterior circulation (p=1.0) reached statistical significance.

Conclusion

In this cohort, intervention timing, vascular territory, and patient demographics had no significant impact on complication rates. Technical success of intracranial angioplasty and stenting exceeded 97%, with no association between acute procedures or posterior lesions and poorer outcomes. These null results should be viewed in light of the limited sample size. Overall, endovascular revascularization proved feasible across varied patient subgroups.

## Introduction

Intracranial atherosclerotic disease (ICAD) is a leading cause of ischemic stroke worldwide, with population studies reporting a 5-7% prevalence of asymptomatic intracranial stenosis in adults over 50 [1,2]. Symptomatic ICAD is particularly concerning, accounting for 30-50% of ischemic strokes in high-risk populations, and is associated with higher stroke recurrence rates compared to other stroke etiologies [1,2]. Epidemiologic data highlight disparities in ICAD burden, with Asian, African American, and Hispanic populations disproportionately affected [2-5].

The optimal management of ICAD remains ambiguous. Early randomized trials, such as the Warfarin versus Aspirin for Symptomatic Intracranial Disease (WASID) trial, demonstrated that medical therapy alone yielded suboptimal outcomes, with a 12% annual stroke risk in high-grade stenosis (>70%) [6]. The subsequent Stenting and Aggressive Medical Management for Preventing Recurrent Stroke in Intracranial Stenosis (SAMMPRIS) trial further challenged endovascular approaches, showing a 14.7% 30-day stroke/death rate with stenting versus 5.8% with medical therapy [7]. However, SAMMPRIS's use of off-label stent use and early intervention within eight days of symptoms prompted reevaluation [8,9].

The Wingspan Stent System Post Market Surveillance (WEAVE) trial, which adhered to strict on-label patient selection and a ≥7-day delay from symptom onset, reported a 2.6% periprocedural complication rate [9]. One-year follow-up data from the Wingspan One-year Vascular Events and Neurologic Outcomes (WOVEN) trial further supported durability, with an 8.5% stroke/death rate at one year [10]. These findings underscore the potential roles of operator experience, patient selection, and procedural timing in optimizing outcomes.

Despite these advances, critical questions remain unresolved. Does hyperacute stenting (≤7 days) independently increase complications? Are posterior circulation lesions at higher risk for poor outcomes? Can antiplatelet response testing mitigate periprocedural risks?

To address these gaps, we analyzed 37 consecutive patients undergoing angioplasty±stenting for symptomatic ICAD, stratified by acute (≤7 days) versus subacute (>7 days) intervention. We evaluated 72-hour and 30-day complications, based on angioplasty±stenting vs. angioplasty alone.

## Materials and methods

Study design, setting, and population

This retrospective study evaluated outcomes in patients with intracranial stenosis who underwent either angioplasty alone using the Gateway balloon (Stryker, Kalamazoo, Michigan, United States) or angioplasty with stent placement. The primary stent system employed was the Wingspan stent with Gateway angioplasty (Stryker, Kalamazoo, Michigan, United States). In select cases, alternative devices were utilized, including the Rebel coronary balloon-mounted stent (Boston Scientific, Marlborough, United States) or the Neuroform Atlas self-expanding stent (Stryker, Kalamazoo, Michigan, United States) [11-15].

The analysis included 37 patients treated at Prisma Health Midlands, between 2011 and 2020, categorized into two groups based on intervention timing: acute (≤7 days from symptom presentation) and subacute (>7 days from presentation). Inclusion criteria were patients with symptomatic focal ischemia or transient ischemic attack (TIA) attributed to a 70-99% intracranial atherosclerotic lesion confirmed by angiographic imaging, and exclusion criteria were non-atherosclerotic stenosis. All interventions were conducted under general anesthesia. A standardized antiplatelet regimen was implemented based on intervention timing: patients undergoing acute stenting during mechanical thrombectomy received a loading dose of clopidogrel (600 mg) and aspirin (650 mg) via nasogastric tube upon stent decision, while non-acute cases received pretreatment with clopidogrel (75 mg daily) or aspirin (325 mg daily) for five days preceding the procedure [11-14,16].

The transfemoral approach utilized an 8F sheath with an 8F guide catheter and 6F intermediate catheter system (Cook Medical, Bloomington, Indiana, United States). To accommodate the Wingspan stent system's 135 cm delivery platform, guide catheters were optimized for length while maintaining access stability, supplemented by short Tuohy-Borst adapters (Cook Medical, Bloomington, Indiana, United States) when necessary [17]. Lesion crossing was achieved using microcatheter/microwire techniques followed by Gateway balloon exchange. Balloon sizing was standardized at 80% of the native vessel diameter, with controlled inflation at 1 atmosphere per minute. Precise Wingspan stent deployment centered on the lesion was performed without post-stent angioplasty. All patients were monitored post-procedure in a neurointensive care unit with strict blood pressure monitoring.

Data analysis

We constructed separate tables categorizing patients by intervention (stent vs. angioplasty‑only), outcome (complication vs. no complication), demographics (female vs. male, age), ischemic location (posterior circulation vs. anterior circulation), and timing (acute vs. subacute) to calculate odds ratios (OR) and p‑values. The OR for each comparison was calculated as the ratio of the odds of the event in the first subgroup to the odds in the second subgroup [18]. Due to several cells having small sample sizes, we used a two‑sided Fisher's exact test to compute p‑values for each table [19]. For the demographic table, mean age and standard deviation were first computed for each subgroup, and then the 30‑day complication/death counts were dichotomized, from which the OR and Fisher's exact p‑value were derived. This approach ensures accurate inference despite the modest sample size and sparse data [19].

Ethical clearance

Prisma Health IRB issued approval. A document demonstrating this study has been provided exemption.

## Results

Timing and location of intervention 

Table 1 demonstrates that 86% of patients underwent angioplasty with stent placement, while 14% of patients had angioplasty alone. There were no statistically significant differences between intervention types in timing (acute vs. subacute) or circulation territory (all p≥0.31).

**Table 1:** Treatment modality, location, and clinical presentation timing Significant level at p<0.05

Intervention	Angioplasty and stent (n=32)	Angioplasty only (n=5)	P-value
Patients (total n=37)	32 (86%)	5 (14%)	-
Acute (<7d)	23 (72%)	5 (100%)	0.31
Subacute (≥7d)	9 (28%)	0 (0%)	0.31
Anterior circulation	14 (44%)	2 (40%)	1
Posterior circulation	18 (56%)	3 (60%)	1

Complications with intervention 

Table 2 compares the odds of early (≤ 72-hour) and delayed (30-day) complications between patients undergoing angioplasty with stent placement and angioplasty alone. None of the OR reached statistical significance (all p>0.05). 

**Table 2:** Complication rates at 72 hours and 30 days by treatment modality Significant level at p<0.05

Intervention	Angioplasty and stent (n=32)	Angioplasty only (n=5)	Odds ratio	P‑value
Complications	6 (19%)	2 (40%)	0.35	0.29
72 hours	5 (83%)	1 (50%)	0.74	1
30 days	1 (17%)	1 (50%)	0.13	0.25

Patient demographics

Table 3 contains a summary of demographic subgroups and 30‑day complication/death outcomes. It also presents the mean age and standard deviation (SD) for female versus male patients and for patients below versus above 64 years, along with the OR and p‑value for 30‑day complication or death comparing each first subcategory to its counterpart. The analysis demonstrates no statistically significant difference in 30‑day adverse events between genders or age groups (all p>0.05).

**Table 3:** Patient demographics and statistical analysis of 30-day complication/death Significant level at p<0.05

Category	Subcategory	Mean age (years)	Standard deviation of mean age (years)	Odds ratio (30‑day complication/death)	P‑value
Gender	Female	66.1	16.4	0.42	0.67
	Male	63.56	13.64		
Age	Below 65	52.19	7.43	2.19	0.36

## Discussion

In this retrospective series of 37 patients, no statistically significant predictors of periprocedural complications emerged. Angioplasty with stent versus angioplasty alone showed no significant difference in early (≤72-hour) or 30-day events (Table 2). Likewise, patient age and sex bore no relation to 30-day outcome (Table 3). Although numerically all complications occurred in the acute (≤7 days) treatment group (eight of 28 patients) versus none in the subacute (>7 days) group (Table 1), this difference did not reach statistical significance (all p>0.05).

We asked whether stenting in the acute vs. subacute setting increased complications. In our cohort, 8/28 (28.6%) acute patients had complications versus 0/9 subacute patients. However, Fisher's exact test for this comparison was not significant (p=0.16). While acute cases accounted for all observed events, the sample was too small to conclude that acute timing caused a higher risk. This is consistent with the data in Table 2, which show no significant difference in any complication endpoint (all p>0.05). Our data does not by itself prove a causal link between timing and complications. However, a retrospective study performed with 96 patients found statistically significant favorable outcomes with stenting after acute occlusive stroke, corroborating the trend of our study with angioplasty with stent being favorable to angioplasty alone (Table 2) [20]. Furthermore, as our study found age and sex to be independent factors in predicting outcomes (Table 3) (all p>0.05), other literature that expands the follow-up window for all-cause mortality beyond 30 days found statistically significant worse outcomes for those aged 61 and older [20,21].

We also asked if posterior circulation lesions carried a higher risk. Overall event numbers were low, and the difference in complication rates by location was not statistically significant (Table 1; p=1). We did not find posterior location to be an independent predictor of complications in our analysis. Our results (all p>0.05) do not prove that location alone drives adverse events, which is in line with the literature indicating that patients' post-stroke mechanical thrombectomy have no statistically significant difference in functional outcome at 90 days [22].

Important limitations must be acknowledged when interpreting these results. The relatively small sample size (n=37) limits the statistical power for subgroup analyses and multivariate modeling of risk factors. Additionally, the lack of long-term follow-up data beyond 30 days prevents the assessment of durable treatment effects and restenosis rates. These limitations highlight the need for larger, prospective studies with standardized protocols and extended follow-up periods.

## Conclusions

Our analyses revealed no statistically significant differences by timing, lesion site, or patient demographics. Thus, while we observed that the technically successful revascularization rate was high, no clear signal emerged linking acute intervention or posterior location to poorer outcomes in this cohort. Given the small sample size, these null findings must be interpreted cautiously. Our results reinforce that intracranial angioplasty and stenting can be performed with high technical success and independent of sex.
